# The changing landscape of research funding: challenges for mid-career researchers

**DOI:** 10.1186/s13059-019-1798-9

**Published:** 2019-08-28

**Authors:** Nick Wong

**Affiliations:** Monash Bioinformatics Platform Central Clinical School, Monash University, The Alfred Research Alliance, 55 Commercial Road, Melbourne, VIC 3004 Australia

## Abstract

Funding research is a challenge faced by most scientists around the world. Genome Biology has invited four scientists based in three different countries to share their own experience and opinions regarding funding, the difficulties young scientists must overcome, and how the process of securing funding can be improved. In this part, Nick Wong shares his experience in securing funding for his research in Australia.

## Main text

I am currently a Senior Research Fellow within the Monash Bioinformatics Platform at Monash University. The Platform is one of 25 research platforms that Monash University have established to support academic research. While there are opportunities to drive my own research, this is not a priority in my current role. Other research platforms are mainly infrastructure-based, such as confocal microscopy and imaging, and antibody production.

My academic research career began with obtaining a PhD at the University of Melbourne in 2006. This was at a time when the overall success rate for gaining National Health and Medical Research Council (NHMRC) project grants, Australia’s peak funding body for medical research, was about 30% and corresponded to the funding of one in three grants submitted by the laboratory group of which I was a part.

During my postdoctoral training, I was very fortunate to be supported by the Leukemia Foundation of Australia in the form of a postdoctoral research fellowship and a Grant-In-Aid project grant towards establishing my own research effort into the role of DNA methylation in pediatric leukemia. Fresh from a PhD, junior postdoctoral positions are relatively straightforward to come by, with many laboratory groups at the time having spare funds for new postdoctoral positions. Securing a training fellowship with the NHMRC in the first years of postdoctoral training was a challenge but not out of reach. One’s publication track record was evaluated but also the track record of the host laboratory group, whether a local research group with national recognition or an international group with international standing. I chose to stay in Australia rather than move further abroad for experience because I am from New Zealand originally, so I was already outside my home country.

As I progressed through my postdoctoral training, the funding success rate plummeted, and fellowships were more out of reach. The large emphasis on past performance (publication track record) as a measure of future performance was engrained by most anonymous peer-review comments that were appraising the project and for fellowship applications. In Australia, the differentiation between an early career researcher to a mid-career researcher is 2 years of postdoctoral experience. Securing funding as a mid-career researcher was progressively challenging. There were two levels of mid-career funding available, the first for applicants with 2–7 years postdoctoral experience and the second for applicants with 7–12 years postdoctoral experience. Given the emphasis on publication track record, these seemingly arbitrary lines made applicants at the junior end of both spectrums disadvantaged compared with applicants at the experienced end.

As with project grants, fellowship applications relied heavily on the publication track record as a measure of future performance—not only authorship position, but also in which journal your work was published. I was fortunate to be part of a group in which the laboratory heads were open to fostering my career, allowing me senior authorship from my projects. Successful funding rates plummeted to 17% at one stage, which was confounded by an increase in applications to the NHMRC and an effective funding freeze from Federal Government.

This was a very low point in my career, and I started to consider moving to industry, which at the time, in my mind, was considered a cop-out. The culture was such that if you have a PhD, your vocation was academic research; anything else was second best. I was fortunate to have career coaching during this low point whereby I came to realize that the skill sets I had were attractive to industry. Linking up with industry workers, I found it was the best thing they ever did—leaving academia and never looking back—a concept that was rather foreign to me. The tipping point was from a senior academic, a leader in their field in Australia, who said to me that moving to industry does not mean you cannot come back to academia. In fact, the industry experience they acquired was a large contributing factor to their success in academia today. This was the same person who, at the time I was applying for mid-career fellowships, was very worried whether their fellowship application with the NHMRC would be successful.

On moving to industry, most aspects I expected to encounter played out. Having an ongoing role was nice compared with the 12-month rolling contract review and renewal in academia. Funding was from a different source. However, accounting for and justifying the spending on projects was, in my opinion, much more rigorous. The peer reviewers were internal; colleagues were from a wider experience domain. There were no set dates for application submissions, if a project needed to be done, it was written up, reviewed and considered in a short time span. The stakeholders were closer to home compared with academic grant bodies. The science was very relevant and essential for commercial translation; however, it would not have been funded in an academic setting.

I am now back in an academic role and am more resilient having spanned both the so-called light side and dark side of science. The academic funding landscape in medical research is evolving in Australia [1]. It is hoped that the changes provide tangible opportunities for researchers across all career stages, including those like me. Submissions to the new system are open, with funding commencing in 2020. There is a sense of hope from many colleagues regarding the impact of these changes. It is tempered with a dash of uncertainty across the sector, where it is a challenge for universities and academic research institutions to model and forecast future research income. Nevertheless, I will be throwing my hat in the ring through a scheme in which the publication record of the applicant is not a large component.
